# *Bemisia tabaci* Vesicle-Associated Membrane Protein 2 Interacts with Begomoviruses and Plays a Role in Virus Acquisition

**DOI:** 10.3390/cells10071700

**Published:** 2021-07-05

**Authors:** Yun-Yun Fan, Yu-Wei Zhong, Jing Zhao, Yao Chi, Sophie Bouvaine, Shu-Sheng Liu, Susan E. Seal, Xiao-Wei Wang

**Affiliations:** 1 Ministry of Agriculture Key Laboratory of Molecular Biology of Crop Pathogens and Insects, Institute of Insect Sciences, Zhejiang University, Hangzhou 310058, China; yyfan@zju.edu.cn (Y.-Y.F.); ywzhong@zju.edu.cn (Y.-W.Z.); swuzhj19940215@163.com (J.Z.); 21616153@zju.edu.cn (Y.C.); shshliu@zju.edu.cn (S.-S.L.); 2 Natural Resources Institute, University of Greenwich, Chatham, Kent ME4 4TB, UK; s.bouvaine@greenwich.ac.uk

**Keywords:** acquisition, begomovirus, coat proteins, SLCMV, TYLCV, transmission, VAMP2, whitefly

## Abstract

Begomoviruses cause substantial losses to agricultural production, especially in tropical and subtropical regions, and are exclusively transmitted by members of the whitefly *Bemisia tabaci* species complex. However, the molecular mechanisms underlying the transmission of begomoviruses by their whitefly vector are not clear. In this study, we found that *B. tabaci* vesicle-associated membrane protein 2 (BtVAMP2) interacts with the coat protein (CP) of tomato yellow leaf curl virus (TYLCV), an emergent begomovirus that seriously impacts tomato production globally. After infection with TYLCV, the transcription of *BtVAMP2* was increased. When the BtVAMP2 protein was blocked by feeding with a specific BtVAMP2 antibody, the quantity of TYLCV in *B. tabaci* whole body was significantly reduced. BtVAMP2 was found to be conserved among the *B. tabaci* species complex and also interacts with the CP of Sri Lankan cassava mosaic virus (SLCMV). When feeding with BtVAMP2 antibody, the acquisition quantity of SLCMV in whitefly whole body was also decreased significantly. Overall, our results demonstrate that BtVAMP2 interacts with the CP of begomoviruses and promotes their acquisition by whitefly.

## 1. Introduction

Geminiviruses (*Geminiviridae*) are a group of plant viruses characterized by a genome of circular single-stranded DNA encapsidated in twinned virions. These viruses are distributed in tropical and subtropical regions and cause economically important diseases to food and fiber crops worldwide [1,2,3]. The family *Geminiviridae* is classified into nine genera of which the genus *Begomovirus* is the largest, including 424 species to date [4,5]. Two begomoviruses are listed in the top 10 plant viruses of scientific/economic importance globally, namely, tomato yellow leaf curl virus (TYLCV) and African cassava mosaic virus (ACMV) [6]. TYLCV has quickly spread from the Middle East to the rest of the world from the early 1960s with devastating economic losses to tomato production [7]. Cassava (*Manihot esculenta* Crantz, Euphorbiaceae) is a staple food of nearly a billion people in 105 countries [8]. Cassava mosaic disease (CMD) is the main biotic and economically important constraint on cassava cultivation. ACMV and a further nine cassava mosaic begomoviruses (CMBs) in Africa, as well as Indian cassava mosaic virus (ICMV) and Sri Lankan cassava mosaic virus (SLCMV) in Asia, are the causal agents of CMD [3,9]. In the past few years, similar to the invasive spread of TYLCV, SLCMV has rapidly invaded Cambodia, Vietnam, and China, causing great losses to the cassava industry [10,11,12].

Begomoviruses are exclusively transmitted by the whitefly *Bemisia tabaci* (Gennadius) in a persistent circulative manner. Once acquired orally by whiteflies, begomoviruses follow the sequential path of stylet–midgut–haemolymph before entering the salivary glands; finally, viruses are delivered into plants with the secretion of saliva during the next feeding [13,14]. It is worth noting that *B. tabaci* is a species complex containing at least 44 cryptic species with distinct genetic structure and biological traits but indistinguishable external morphology [15,16]. Different cryptic species of whitefly have different transmission efficiencies of a given begomovirus, and different viruses may be transmitted with contrasting efficiencies by a given whitefly species [17]. Among these, the invasive Middle East-Asia Minor 1 (MEAM1) efficiently transmits TYLCV but not SLCMV [18,19,20]. In contrast, an indigenous *B. tabaci* species Asia II 1 efficiently transmits SLCMV and poses a potential threat to cassava production in many regions [20].

The successful transmission of begomoviruses by *B. tabaci* depends on the interaction of proteins between the virus and the whitefly vector. Recently, several proteins of *B. tabaci* have been identified that affect the acquisition and transmission of begomoviruses [21]. The heat shock protein 70 (HSP70) and vesicle-associated membrane protein-associated protein B (VAPB) negatively regulate the acquisition and transmission of viruses [22,23], while midgut protein (MGP), cyclophilin B (CyPB), and collagen proteins play a positive role [24,25,26]. Previous studies have shown that begomoviruses are delivered into the midgut cells of *B. tabaci* via clathrin-mediated endocytosis where they are encapsulated in vesicle structures [27,28,29]. Subsequently, Zhao et al. [30] found that cubilin (CUBN) and amnionless (AMN) form the endocytosis receptor complex BtCubam in the *B. tabaci* midgut. TYLCV CP could bind to BtCubam through the interaction with a specific structure domain of BtCUBN, to facilitate the transport across the midgut barrier via clathrin-mediated endocytosis. However, although vesicle structure is important for virus acquisition and transmission, no vesicle protein has been reported to date that takes part in begomovirus transmission.

The coat protein (CP) of a begomovirus is the only protein that is known to be involved in its transmission by vector(s) in the *B. tabaci* complex [31,32,33,34]. In this study, we used co-immunoprecipitation (Co-IP) followed by LC-MS/MS analysis to identify the whitefly interactors of TYLCV CP, and selected one protein, namely, vesicle-associated membrane protein 2 (VAMP2) for further analysis, as it is involved in vesicle-mediated transport pathways [35,36]. Using biological and molecular assays, including Co-IP, glutathione S-transferase (GST)-pull down, gene expression analysis, and virus acquisition and transmission, we found that BtVAMP2 interacts with the CP of begomoviruses including TYLCV and SLCMV, and supports virus acquisition. Our findings provide new insights for further understanding the interaction mechanisms between *B. tabaci* and begomoviruses.

## 2. Materials and Methods

### 2.1. Plants, Insects, and Viruses

The plants used were cotton (*Gossypium hirsutum* L. cv. Zhemian 1793), tobacco (*Nicotiana tabacum* L. cv. NC89), and tomato (*Solanum lycopersicon* L. cv. Hezuo903). All plants were planted in a greenhouse under natural lighting and maintained under controlled conditions of 25 ± 3 °C, 14 h light/10 h darkness.

Whitefly *B. tabaci* MEAM1 (mt*COI* GenBank accession number: GQ332577) and Asia II 1 (mt*COI* GenBank accession number: DQ309077) were cultured in cages in an artificial climate chamber on cotton at 26 ± 1 °C, 60% relative humidity, and a cycle of 14 h light/10 h darkness. The purity of the whitefly population was analyzed by mt*COI* PCR-RFLP and sequencing according to Qin et al. [37].

An infectious clone of TYLCV-SH2 (GenBank accession: AM282874) was provided by the Institute of Biotechnology of Zhejiang University, and the construction method of infectious clones of SLCMV (GenBank accession: KT861468 for DNA-A and KT861469 for DNA-B) was as previously reported [20]. TYLCV or SLCMV infectious clones were inoculated to 2–3 true leaf stage tomato seedlings or 4–5 true leaf stage tobacco plants, respectively, to obtain infected plants. About 4 weeks later, DNA extraction was performed using the Plant Genomic DNA Kit (Tiangen, Beijing, China), and then virus infections were detected in plants by PCR using primers TYLCV-PCR-F (5′-GTCGAAGCGACCAGGCGATA-3′), TYLCV-PCR-R (5′-GGTTCTCATACTTGGCTGCCTCC-3′), SLCMV-PCR-F (5′-CAGCAGTCGTGCTGCTGTC-3′), and SLCMV-PCR-R (5′-TGCTCGCATACTGACCACCA-3′).

### 2.2. Co-Immunoprecipitation (Co-IP) Analysis

After a 7 d acquisition on virus-infected plants, the proteins of *B. tabaci* were extracted using cytoplasmic extraction buffer (Invent, Beijing, China). Then, anti-TYLCV CP mouse monoclonal antibody (mAb) (kindly provided by Professor Wu Jianxiang, Institute of Biotechnology, Zhejiang University) was added into the cytoplasmic proteins at a 1:100 (*v*/*v*) dilution and incubated overnight at 4 °C. Controls included were pre-immune serum (Beyotime, Shanghai, China) or proteins of uninfected whiteflies. Protein G-Sepharose beads (GE Healthcare, Boston, MA, USA) were subsequently added and the mixture incubated at 4 °C for 4 h. The beads were then washed five times with 1x PBS. To elute co-immunoprecipitated proteins, beads were boiled in SDS-PAGE buffer (FDBIO, Hangzhou, China) for 10 min. Finally, the proteins were subjected to SDS-PAGE electrophoresis and the resulting proteins then processed by LC-MS/MS, or by Western blot with anti-BtVAMP2 rabbit polyclonal antibody (pAb), which was produced by GenScript (Nanjing, China) using synthetic peptides (DSGANLSTGEDGIVG) conjugated to keyhole limpet hemocyanin as antigens. Specificity of the generated anti-BtVAMP2 antibody was tested by Western blot and shown in Supplementary Figure S1.

### 2.3. LC-MS/MS Analysis

After Co-IP, Coomassie blue staining, and decoloring procedures, the protein gels were cut down and an LC-MS/MS assay was performed as described previously [30,38]. Finally, the captured peptides were annotated according to the MEAM1 protein sequence information (http://www.whiteflygenomics.org, accessed on 20 January 2020).

### 2.4. GST Pull-Down Assay

TYLCV CP and SLCMV CP genes were cloned into pGEX-6p-1 for fusion with glutathione S-transferase (GST), respectively, with primers TYLCV CP-pGEX-6p-1-F (5′-CGCGGATCCATGTCGAAGCGACCAGG-3′), TYLCV CP-pGEX-6p-1-R (5′-CCGGAATTCTTAATTTGATATTGAATCATA-3′), SLCMV CP-pGEX-6p-1-F (5′-CGCGGATCCATGTCGAAGCGACCAGCAGA-3′), and SLCMV CP-pGEX-6p-1-R (5′-CCGGAATTCTTAATTGCTGACCGAATCGT-3′). The recombinant prokaryotic expression vector or no load (negative control) were transferred into *E. coli* strain BL21 for expression. The expressed proteins were combined with glutathione agarose beads (GE Healthcare, Boston, MA, USA) at 4 °C for 2 h. The beads were then washed five times with 1xPBS in 6 mL affinity chromatography columns (Sangon, Shanghai, China). Then, the GST or GST-CP proteins were analyzed by SDS-PAGE electrophoresis and Coomassie blue staining assay after boiling in SDS-PAGE buffer for 10 min. After that, the cytoplasmic proteins of whitefly were added to the beads-bound GST or GST-CP proteins as prey proteins at 4 °C for 4 h. Next, these mixtures were centrifuged and washed with 1xPBS five times, and then the beads-bound proteins were eluted by boiling in SDS-PAGE buffer for 10 min. Finally, the proteins were separated by SDS-PAGE electrophoresis and detected by anti-BtVAMP2 antibody.

### 2.5. Virus Quantity and Gene Transcription Analysis

*B. tabaci* were fed on virus-infected plants (or uninfected plants as a negative control) for 0, 24, 48, and 96 h. At each time point, ten females were collected for DNA extraction following the methods of Pan et al. [28] to quantity the virus in *B. tabaci* by quantitative real-time PCR (qPCR). The primers used for TYLCV and SLCMV quantification were TYLCV-qPCR-F (5′-GAAGCGACCAGGCGATATAA-3′), TYLCV-qPCR-R (5′-GGAACATCAGGGCTTCGATA-3′), SLCMV-qPCR-F (5′-ACGCCAGGTCTGAGGCTGTA-3′), and SLCMV-qPCR-R (5′-GTTCAACAGGCCGTGGGACA-3′). Additionally, at each time point, thirty whiteflies were collected for extraction of total RNA using TRIzol (Ambion, Waltham, MA, USA), and EVO-M-MLV reverse transcription kit II (Accurate Biology, Changsha, China) for cDNA synthesis. Finally, qPCR was used to analyze the BtVAMP2 transcription using primers VAMP2-qPCR-F (5′-TGCTTAGCATTGGGAGTTGC-3′) and VAMP2-qPCR-R (5′-GTCTGGTGCTGCCATTCTTT-3′). For each analysis, 3–4 replicates were conducted. All qPCR detections were performed using SYBR Green Premix Pro Taq HS qPCR Kit (Accurate Biology, Changsha, China) and the CFX96 Real-time PCR Detection System (Bio-Rad, Hercules, CA, USA). *β-actin* of *B. tabaci* (β-actin-qPCR-F 5′-TCTTCCAGCCATCCTTCTTG-3′ and β-actin-qPCR-R 5′-CGGTGATTTCCTTCTGCATT-3′) was used as the reference gene for transcript and virus quantification.

### 2.6. Membrane Feeding of Antibody

The whitefly adults were collected and released into glass tubes of which one end was covered with a double layer of parafilm filled with antibody and the other end with a layer of gauze [28]. BtVAMP2 antibody (0.713 mg/mL) was added into 15% sucrose at a 1:50 (*v*/*v*) dilution. At the same time, rabbit pre-immune serum (diluted to 0.713 mg/mL) was added into 15% sucrose as a negative control. Whiteflies were left to feed on the antibody and control solutions for 24 h.

### 2.7. Quantification of Virus in Whitefly after Feeding Antibody

After feeding antibody or pre-immune serum for 24 h, *B. tabaci* were transferred to infected plants for virus acquisition. After 48 or 96 h, 10 females were collected per sample for DNA extraction and virus quantification as described above.

### 2.8. Virus Transmission

After 24 h antibody feeding and 48 h TYLCV acquisition, 4 (female/male = 1:1) MEAM1 adults were transferred in a clip-cage [39] onto a 2–3 true-leaf stage tomato seedling to feed for 48 h. For transmission of SLCMV, after a 96 h acquisition period, 10 females of Asia II 1 were collected and placed on a 4–5 true-leaf stage tobacco seedling for 96 h. After removing the adult whiteflies, the plants were sprayed with a 500× diluted solution of 20% acetamiprid to kill the eggs. After four weeks, the status of plant infection was determined by PCR detection of viral DNAs, as described above. Each treatment was repeated three times, and each replicate consisted of 7–8 plants.

### 2.9. Statistical Analysis

All qPCR data were calculated using 2^−ΔCT^ as normalized to whitefly *β-actin*. For comparing the effect of feeding anti-BtVAMP2 antibody on virus acquisition, the quantity of virus in the whole body of whiteflies feeding with pre-immune serum was set to 1. Statistical analyses were conducted using IBM SPSS Statistics 20 software (IBM, Armonk, NY, USA), and all data were analyzed by independent *t*-test (statistically significant differences are indicated as: * *p* < 0.05, ** *p* < 0.01, and *** *p* < 0.001). For the comparison of transmission efficiency, percentage data were arcsine square root transformed for statistical analysis, and the data presented in the figure are raw data.

## 3. Results

### 3.1. Interactions between TYLCV CP and BtVAMP2

**Figure 1:**
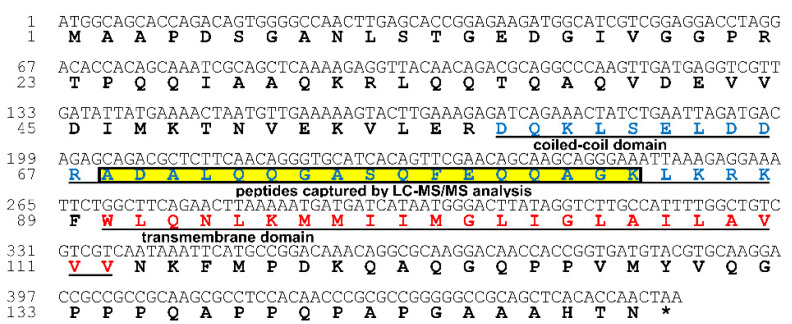
The deduced amino acid sequence of MEAM1 VAMP2. The underlined blue amino acids indicate a coiled coil domain; the underlined red amino acids indicate a transmembrane domain. The yellow boxed sequence represents the peptides captured by Co-IP coupled with LC-MS/MS assays.

Previous studies have shown that, during transportation in their vectors, begomoviruses were enclosed in vesicles [28,30]. To identify the putative proteins that bind to the TYLCV CP in the MEAM1 whitefly, we conducted Co-IP (Supplementary Figure S2) coupled with LC-MS/MS assay, and the proteins were listed in Supplementary Table S1. Among the candidate proteins identified, vesicle-associated membrane protein 2 (BtVAMP2, NCBI accession number: XP_018904064.1) was subjected to further analysis as it is annotated to the vesicle-mediated transport pathway (GO:0016192) by gene ontology analysis. However, it is hard to identify the band of BtVAMP2 in the gel picture (Supplementary Figure S2) after Co-IP analysis via Coomassie blue staining. As the BtVAMP2 on the gel picture should be close to 16 kDa, it is difficult to visualize in this gel picture. BtVAMP2 contains a coiled coil domain (58–88 aa), a transmembrane region (90–112 aa), and the peptides of BtVAMP2 captured by Co-IP coupled with LC-MS/MS were located in the 68–84 aa region (Figure 1).

**Figure 2:**
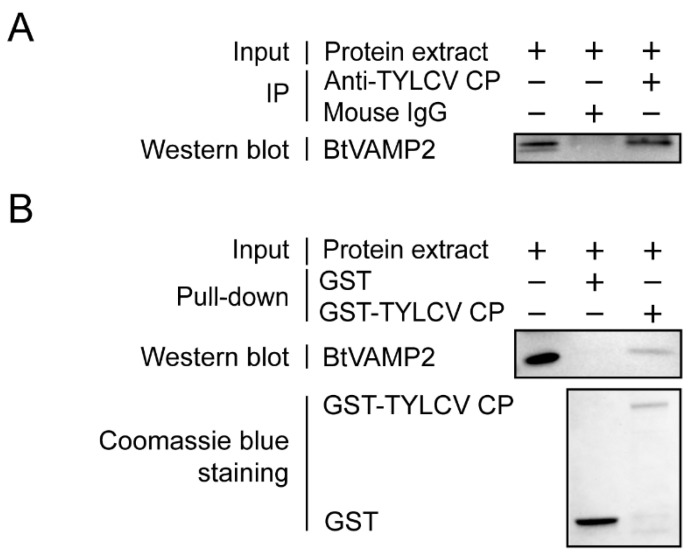
Interaction analysis between BtVAMP2 of MEAM1 whitefly and TYLCV CP. (**A**) Co-IP assay. Cytoplasmic proteins of MEAM1 were extracted following a 7 d virus acquisition and then the proteins were incubated with either anti-TYLCV CP mouse mAb and protein G-Sepharose beads or mouse IgG and protein G-Sepharose beads as a negative control. After Co-IP, anti-BtVAMP2 rabbit pAb was used to detect BtVAMP2 by Western blot. (**B**) GST pull-down analysis. GST-TYLCV CP or GST proteins were expressed and combined with the glutathione Sepharose beads, before being added to the native cytoplasmic protein of MEAM1. Finally, anti-BtVAMP2 rabbit pAb was used to detect whether the BtVAMP2 was pulled down.

Next, we conducted Co-IP, GST pull-down assays to examine further the interaction between BtVAMP2 and TYLCV CP. Co-IP assay showed BtVAMP2 interacted with TYLCV CP in vivo (Figure 2A), and GST pull-down assay showed that BtVAMP2 could be pulled down by GST-TYLCV CP (Figure 2B).

### 3.2. Transcription of BtVAMP2 upon TYLCV Infections

**Figure 3:**
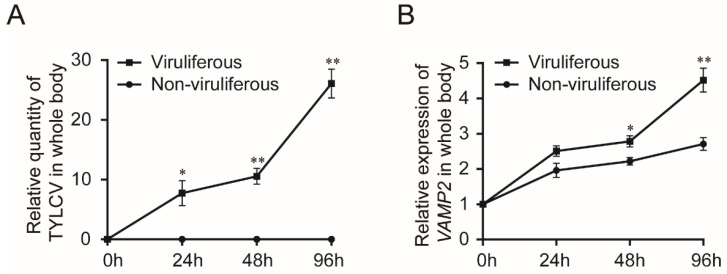
Effect of TYLCV-infections on the transcription of *BtVAMP2*. (**A**) Relative quantity of TYLCV in whitefly after different acquisition periods. (**B**) Relative transcription level of *BtVAMP2* at different acquisition periods in MEAM1 whiteflies fed on TYLCV-infected or uninfected plants (independent *t*-test, *n* = 4, * *p* < 0.05, ** *p* < 0.01).

To explore whether the transcription of BtVAMP2 was affected by TYLCV infection, the quantity of virus and the transcription level of *BtVAMP2* in the whole body were detected by qPCR. According to the results, with the increase of TYLCV quantity in whiteflies (Figure 3A), the transcription of BtVAMP2 also gradually increased; BtVAMP2 transcription levels were significantly higher statistically in TYLCV-viruliferous than non-viruliferous whiteflies at 48 and 96 h (Figure 3B).

### 3.3. Effect of Interfering BtVAMP2 on Virus Acquisition and Transmission

**Figure 4:**
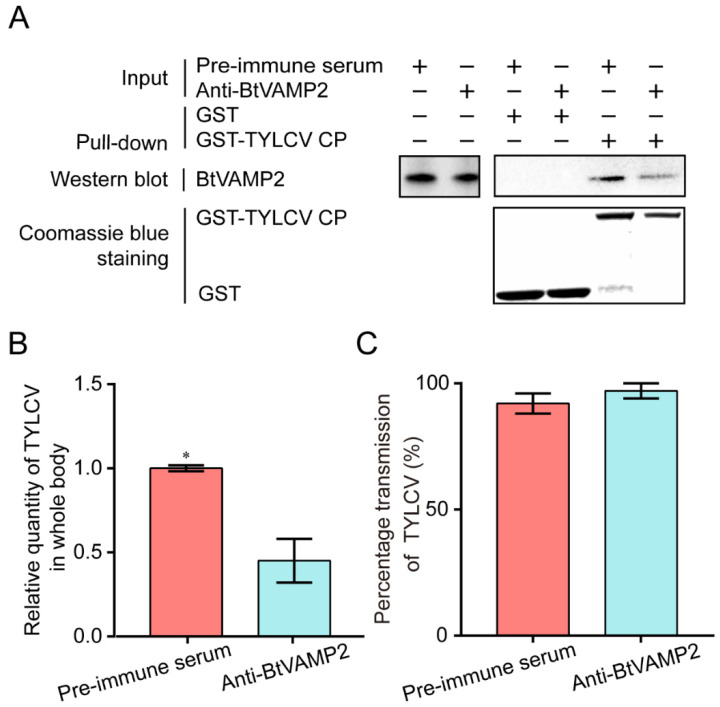
Analyses of the virus quantity in whitefly and the transmission efficiency of TYLCV by whiteflies after feeding with anti-BtVAMP2 pAb. (**A**) GST pull-down analysis shows that the interaction between TYLCV CP and BtVAMP2 was reduced after feeding with anti-BtVAMP2 pAb. (**B**) The TYLCV quantity was detected by qPCR after a 24 h antibody feeding and 48 h virus acquisition (*n* = 3). (**C**) Four whiteflies (female/male = 1:1) were collected and their virus transmission ability was measured (independent *t*-test for (**B**,**C**), *n* = 3, * *p* < 0.05).

In order to confirm whether anti-BtVAMP2 antibody could suppress the binding of TYLCV CP to BtVAMP2, MEAM1 whiteflies were allowed to feed on a diet supplemented with BtVAMP2 antibody. The binding of TYLCV CP to BtVAMP2 was weakened as shown by GST-pull down detection after 24 h feeding (Figure 4A). After feeding on the BtVAMP2 antibody and infected plants, the quantity of virus in the whitefly whole body was significantly decreased, indicating the binding between the virus and BtVAMP2 was conducive to the acquisition of the virus (Figure 4B); however, the transmission efficiency showed no difference (Figure 4C).

### 3.4. Verification of Interaction between BtVAMP2 and SLCMV CP

**Figure 5:**
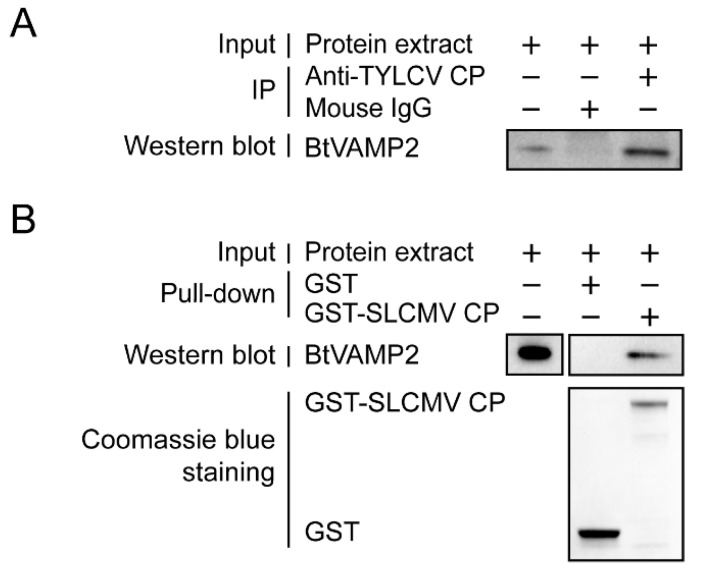
Interaction analysis between Asia II 1 BtVAMP2 and SLCMV CP. (**A**) Co-IP analysis. The cytoplasmic proteins of Asia II 1 whitefly fed on SLCMV-infected plants for 7 d were extracted and incubated with either anti-TYLCV CP mAb and protein G-Sepharose beads, or mouse IgG and protein G-Sepharose beads as a negative control. After Co-IP, anti-BtVAMP2 rabbit pAb was used to detect BtVAMP2 by Western blot. (**B**) GST pull-down analysis. GST-SLCMV CP or GST was used as bait protein, and the native cytoplasmic proteins of Asia II 1 whitefly were used as prey protein. Anti-BtVAMP2 rabbit pAb was used to detect whether BtVAMP2 protein was pulled down.

Next, we compared the amino acid sequences of BtVAMP2 in Asia II 1 VAMP2 and MEAM1. The two sequences from different *B. tabaci* species have a pairwise similarity of 96% (Supplementary Figure S3). We thus examined whether the function of BtVAMP2 in virus acquisition as demonstrated in MEAM1 was conserved in Asia II 1. According to a previous study, Asia II 1 whitefly could effectively transmit SLCMV [20]. We performed Co-IP and GST pull-down assays to examine the interaction between BtVAMP2 of Asia II 1 and SLCMV CP. Our results showed that the two proteins also interacted with each other (Figure 5A,B).

### 3.5. Transcription Level of BtVAMP2 after SLCMV Infection

**Figure 6:**
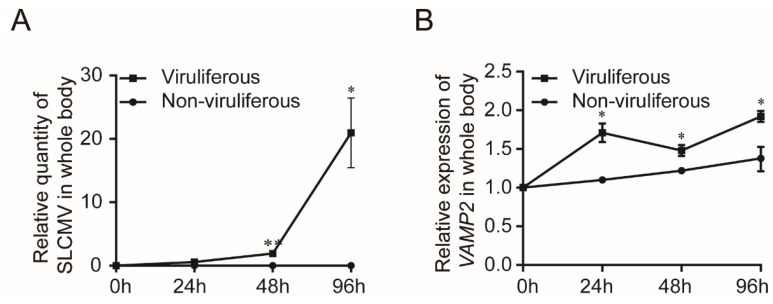
Effect of SLCMV infection on the transcription of *BtVAMP2*. Analyses were conducted with Asia II 1 that fed consecutively either on SLCMV-infected or uninfected plants. (**A**) Relative quantity of SLCMV in whitefly whole body. (**B**) Relative transcription of *BtVAMP2* in whitefly whole body (independent *t*-test for A and B, *n* = 3–4, * *p* < 0.05, * *p* < 0.01).

We detected the quantity of virus and the transcription of *BtVAMP2* in the whole body of Asia II 1 whiteflies after feeding on SLCMV-infected plants. The results showed that the relative quantity of SLCMV was significantly increased after 48 h acquisition (Figure 6A), and the expression of *BtVAMP2* was higher than the one of non-viruliferous whitefly (Figure 6B).

### 3.6. Effects of Interfering Asia II 1 BtVAMP2 Protein on Virus Acquisition and Transmission

**Figure 7:**
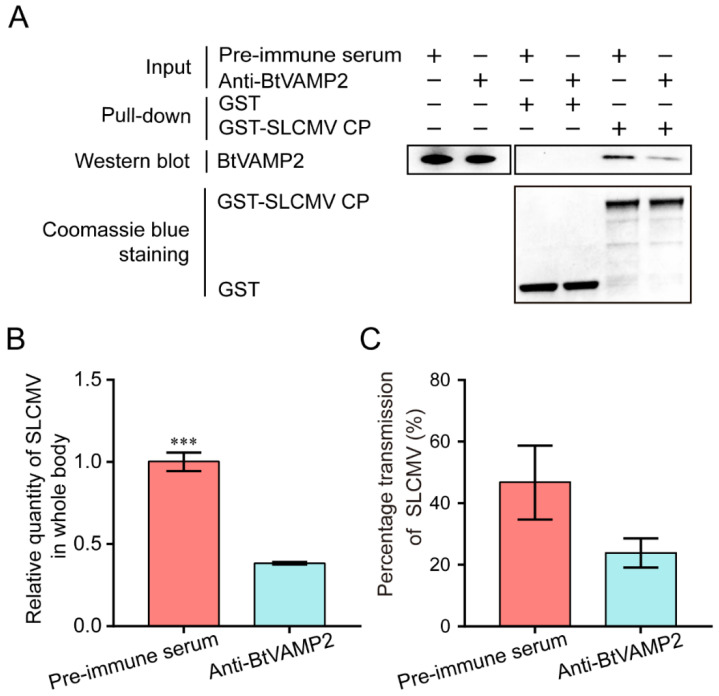
Analyses of the relative quantity of SLCMV in whitefly and the transmission efficiency of SLCMV by whiteflies after antibody treatment. (**A**) GST pull-down analysis shows that the interaction between SLCMV CP and BtVAMP2 was reduced after anti-VAMP2 rabbit pAb-feeding treatment. (**B**) After 24 h antibody feeding and 96 h virus acquisition, SLCMV level in Asia II 1 whole body was analyzed by qPCR. (**C**) Ten female whiteflies were collected and used for virus transmission (independent *t*-test for (**B**,**C**), *n* = 3, *** *p* < 0.01).

After feeding BtVAMP2 antibody for 24 h, the binding of SLCMV CP to BtVAMP2 was weakened as detected by GST-pull down analysis (Figure 7A). The quantity of virus in the whitefly whole body was significantly decreased after interfering with the binding of BtVAMP2 (Figure 7B), but the transmission efficiency showed no significant difference (Figure 7C). These results indicate that the role of BtVAMP2 for virus acquisition appears conserved in different whitefly–begomovirus combinations.

## 4. Discussion

Previous studies have shown that the transport of begomoviruses in the whitefly vector is associated with vesicle trafficking systems, and up to now only VAPB, a vesicle membrane associated protein, has been interpreted as having an inhibiting function on virus transmission [23,27,28,29]. In this study, we demonstrated that protein BtVAMP2, which is an integral component of membranes and predicted to participate in vesicle-mediated transport pathways, could bind to the TYLCV CP (Figure 2A,B). Furthermore, the transcription level of *BtVAMP2* was significantly up-regulated after acquiring the TYLCV virus (Figure 3B), while the quantity of TYLCV in the insect body decreased when binding with BtVAMP2 was blocked by an antibody (Figure 4A,B). These results indicate BtVAMP2 interacts with TYLCV and plays a positive role in virus acquisition. A possible reason for the lack of significant effect on virus transmission (Figure 4C) is that other proteins may play a redundant role with BtVAMP2 in the intracellular transport, therefore interfering with BtVAMP2 alone is not enough to induce a change in transmission efficiency.

Our study showed that Asia II 1 VAMP2 exhibits affinity to SLCMV CP, and its transcription level increased after SLCMV infection (Figure 5and Figure 6). When BtVAMP2 was blocked by a specific antibody, the quantity of virus acquired by Asia II 1 decreased but the virus transmission remained unchanged (Figure 7). These results suggest that the function of BtVAMP2 in different *B. tabaci* cryptic species is conserved for virus acquisition. In recent years, the geographical distribution of SLCMV has been expanding making it a serious threat to cassava cultivation in parts of Asian countries [10,11,12]; however, there are few studies on SLCMV–whitefly interaction. Our study is the first to reveal a transport mechanism of SLCMV in its vector at the whitefly protein level.

SNARE (soluble N-ethylmaleimide-sensitive fusion protein attachment protein receptor) proteins play an indispensable role in the final membrane fusion steps in eukaryotic membrane traffic and autophagy [35,36,40,41,42]. The proposed hypothesis suggests that v-SNAREs (on the vesicles) interact with t-SNAREs (on the target compartment) to form a SNARE complex for membrane fusion. VAMP2 is a v-SNARE protein which with two other t-SNARE proteins, syntaxin 1 and synaptosomal-associated protein 25 kDa (SNAP25), take part in the assembly of a SNARE complex [35,36]. However, it has not been reported that VAMP2 plays a role in virus trafficking in insect vectors to date. In this study, we found that BtVAMP2 positively regulates the quantity of virus in the whitefly body. Previous studies showed BtCubam, a receptor complex, uptakes the virions in the whitefly midgut with the assistance of clathrin-dependent endocytosis [30], and after being delivered into epithelial cells, the virions were wrapped in the vesicle structures and then transferred into the early endosome [29]. We propose that BtVAMP2 might take part in the process of membrane fusion from vesicle to early endosomes to promote the transport of the virus. BtVAMP2 could bind to the virions which were wrapped in the vesicle structures, and then release them into the early endosomes through binding to the t-SNARE proteins on the target membrane. However, the hypothesis remains to be verified, as do the t-SNARE proteins that cooperate with BtVAMP2 on the early endosomes to participate in the intracellular transport of the virus. Transcriptome analysis showed that in whiteflies infected with begomovirus, the differentially expressed genes include those involved in cell cycle, DNA repair, immune responses, and cargo transport [43,44,45]. As a protein on the vesicle membrane that takes part in the transport pathway, *BtVAMP2* is induced during viral infection (Figure 3and Figure 6), which could help facilitate viral transport and the virus to cross the membrane barrier. However, the specific mechanism of how virus infection induces the up-regulation of *BtVAMP2* is still unclear. Whether there are cis elements as well as trans factors that regulate *BtVAMP2* is worthy of further investigation.

To summarize, we have identified an interaction of VAMP2 with the CP of begomoviruses that promotes their acquisition by whitefly. Thus, our findings present a new role of the protein VAMP2 and may facilitate further studies of the numerous other whitefly–begomovirus interactions that lead to devastating crop yield losses globally.

## Data Availability

Data in this study are available from the authors upon request.

## Supplementary Materials

The following are available online at https://www.mdpi.com/article/10.3390/cells10071700/s1, Figure S1: Analysis of anti-BtVAMP2 antibody; Figure S2: The candidate proteins of MEAM1 whitefly that bind to TYLCV CP by Co-IP analysis via Coomassie blue staining; Figure S3: Comparison of BtVAMP2 amino acid sequences of MEAM1 and Asia II 1; Table S1: The candidate proteins of MEAM1 whitefly identified by Co-IP and LC-MS/MS analysis.

## References

[1] Varma A., Malathi V.G. (2003). Emerging geminivirus problems: A serious threat to crop production. Ann. Appl. Biol..

[2] Seal S.E., VandenBosch F., Jeger M.J. (2006). Factors Influencing Begomovirus Evolution and Their Increasing Global Significance: Implications for Sustainable Control. Crit. Rev. Plant Sci..

[3] Rojas M.R., Macedo M.A., Maliano M.R., Soto-Aguilar M., Souza J.O., Briddon R.W., Kenyon L., Rivera-Bustamante R.F., Zerbini F.M., Adkins S. (2018). World Management of Geminiviruses. Annu. Rev. Phytopathol..

[4] Zerbini F.M., Briddon R., Idris A., Martin D.P., Moriones E., Navas-Castillo J., Rivera-Bustamante R., Roumagnac P., Varsani A. (2017). ICTV Virus Taxonomy Profile: Geminiviridae. J. Gen. Virol..

[5] International Committee on Taxonomy of Viruses ICTV. https://talk.ictvonline.org/ictv-reports/ictv_online_report/ssdna-viruses/w/geminiviridae.

[6] Scholthof K.-B.G., Adkins S., Czosnek H., Palukaitis P., Jacquot E., Hohn T., Hohn B., Saunders K., Candresse T., Ahlquist P. (2011). Top 10 plant viruses in molecular plant pathology. Mol. Plant Pathol..

[7] Lefeuvre P., Martin D.P., Harkins G.W., Lemey P., Gray A.J.A., Meredith S., Lakay F., Monjane A., Lett J.-M., Varsani A. (2010). The Spread of Tomato Yellow Leaf Curl Virus from the Middle East to the World. PLoS Pathog..

[8] Cassava for Food and Energy Security. http://www.fao.org/newsroom/en/news/2008/1000899/index.html.

[9] Legg J.P., Kumar P.L., Makeshkumar T., Tripathi L., Ferguson M., Kanju E., Ntawuruhunga P., Cuellar W. (2015). Cassava Virus Diseases. Adv. Appl. Microbiol..

[10] Uke A., Hoat T.X., Quan M.V., Liem N.V., Ugaki M., Natsuaki K.T. (2018). First Report of Sri Lankan Cassava Mosaic Virus Infecting Cassava in Vietnam. Plant Dis..

[11] Wang H.L., Cui X.Y., Wang X.W., Liu S.S., Zhang Z.H., Zhou X.P. (2016). First Report of Sri Lankan cassava mosaic virus Infecting Cassava in Cambodia. Plant Dis..

[12] Wang D., Yao X.M., Huang G.X., Shi T., Wang G.F., Ye J. (2019). First Report of Sri Lankan Cassava Mosaic Virus Infected Cassava in China. Plant Dis..

[13] Hogenhout S.A., Ammar E.-D., Whitfield A.E., Redinbaugh M.G. (2008). Insect Vector Interactions with Persistently Transmitted Viruses. Annu. Rev. Phytopathol..

[14] Czosnek H., Hariton-Shalev A., Sobol I., Gorovits R., Ghanim M. (2017). The Incredible Journey of Begomoviruses in Their Whitefly Vector. Viruses.

[15] Kanakala S., Ghanim M. (2019). Global genetic diversity and geographical distribution of *Bemisia tabaci* and its bacterial endosymbionts. PLoS ONE.

[16] Mugerwa H., Colvin J., Alicai T., Omongo C.A., Kabaalu R., Visendi P., Sseruwagi P., Seal S.E. (2021). Genetic diversity of whitefly (*Bemisia* spp.) on crop and uncultivated plants in Uganda: Implications for the control of this devastating pest species complex in Africa. J. Pest Sci..

[17] Fiallo-Olivé E., Pan L.-L., Liu S.-S., Navas-Castillo J. (2020). Transmission of Begomoviruses and Other Whitefly-Borne Viruses: Dependence on the Vector Species. Phytopathology.

[18] Li M., Hu J., Xu F.-C., Liu S.-S. (2010). Transmission of Tomato Yellow Leaf Curl Virus by two invasive biotypes and a Chinese indigenous biotype of the whitefly *Bemisia tabaci*. Int. J. Pest Manag..

[19] Wei J., Zhao J.-J., Zhang T., Li F.-F., Ghanim M., Zhou X.-P., Ye G.-Y., Liu S.-S., Wang X.-W. (2014). Specific Cells in the Primary Salivary Glands of the Whitefly *Bemisia tabaci* Control Retention and Transmission of Begomoviruses. J. Virol..

[20] Chi Y., Pan L.-L., Bouvaine S., Fan Y.-Y., Liu Y.-Q., Liu S.-S., Seal S., Wang X.-W. (2020). Differential transmission of Sri Lankan cassava mosaic virus by three cryptic species of the whitefly *Bemisia tabaci* complex. Virology.

[21] Wang X.-W., Blanc S. (2021). Insect Transmission of Plant Single-Stranded DNA Viruses. Annu. Rev. Èntomol..

[22] Götz M., Popovski S., Kollenberg M., Gorovits R., Brown J.K., Cicero J.M., Czosnek H., Winter S., Ghanim M. (2012). Implication of *Bemisia tabaci* Heat Shock Protein 70 in Begomovirus-Whitefly Interactions. J. Virol..

[23] Zhao J., Chi Y., Zhang X.-J., Wang X.-W., Liu S.-S. (2019). Implication of whitefly vesicle associated membrane protein-associated protein B in the transmission of Tomato yellow leaf curl virus. Virology.

[24] Rana V.S., Popli S., Saurav G.K., Raina H.S., Chaubey R., Ramamurthy V.V., Rajagopal R. (2015). A *Bemisia tabaci* midgut protein interacts with begomoviruses and plays a role in virus transmission. Cell. Microbiol..

[25] Kanakala S., Ghanim M. (2016). Implication of the Whitefly *Bemisia tabaci* Cyclophilin B Protein in the Transmission of Tomato yellow leaf curl virus. Front. Plant Sci..

[26] Rana V.S., Popli S., Saurav G.K., Raina H.S., Jamwal R., Chaubey R., Ramamurthy V.V., Natarajan K., Rajagopal R. (2019). Implication of the Whitefly, *Bemisia tabaci*, Collagen Protein in Begomoviruses Acquisition and Transmission. Phytopathology.

[27] Uchibori M., Hirata A., Suzuki M., Ugaki M. (2013). Tomato yellow leaf curl virus accumulates in vesicle-like structures in descending and ascending midgut epithelial cells of the vector whitefly, *Bemisia tabaci*, but not in those of nonvector whitefly *Trialeurodes vaporariorum*. J. Gen. Plant Pathol..

[28] Pan L.-L., Chen Q.-F., Zhao J.-J., Guo T., Wang X.-W., Hariton-Shalev A., Czosnek H., Liu S.-S. (2017). Clathrin-mediated endocytosis is involved in Tomato yellow leaf curl virus transport across the midgut barrier of its whitefly vector. Virology.

[29] Xia W.-Q., Liang Y., Chi Y., Pan L.-L., Zhao J., Liu S.-S., Wang X.-W. (2018). Intracellular trafficking of begomoviruses in the midgut cells of their insect vector. PLoS Pathog..

[30] Zhao J., Lei T., Zhang X.-J., Yin T.-Y., Wang X.-W., Liu S.-S. (2020). A vector whitefly endocytic receptor facilitates the entry of begomoviruses into its midgut cells via binding to virion capsid proteins. PLoS Pathog..

[31] Noris E., Vaira A.M., Caciagli P., Masenga V., Gronenborn B., Accotto G.P. (1998). Amino acids in the capsid protein of tomato yellow leaf curl virus that is crucial for systemic infection, particle formation, and insect transmission. J. Virol..

[32] Harrison B., Swanson M., Fargette D. (2002). Begomovirus coat protein: Serology, variation and functions. Physiol. Mol. Plant Pathol..

[33] Guo T., Zhao J., Pan L.-L., Geng L., Lei T., Wang X.-W., Liu S.-S. (2018). The level of midgut penetration of two begomoviruses affects their acquisition and transmission by two species of *Bemisia tabaci*. Virology.

[34] Pan L.-L., Cui X.-Y., Chen Q.-F., Wang X.-W., Liu S.-S. (2018). Cotton Leaf Curl Disease: Which Whitefly Is the Vector?. Phytopathology.

[35] Söllner T., Bennett M.K., Whiteheart S., Scheller R.H., Rothman J.E. (1993). A protein assembly-disassembly pathway in vitro that may correspond to sequential steps of synaptic vesicle docking, activation, and fusion. Cell.

[36] Gerst J.E. (1999). SNAREs and SNARE regulators in membrane fusion and exocytosis. Cell. Mol. Life Sci..

[37] Qin L., Wang J., Bing X.L., Liu S.S. (2013). Identification of nine cryptic species of *Bemisia tabaci* (Hemiptera: Aleyrodidae) from China by using the mtCOI PCR-RFLP technique. Acta Entomol. Sin..

[38] Huang H., Lu J.-B., Li Q., Bao Y.-Y., Zhang C.-X. (2018). Combined transcriptomic/proteomic analysis of salivary gland and secreted saliva in three planthopper species. J. Proteom..

[39] Ruan Y.-M., Luan J.-B., Zang L.-S., Liu S.-S. (2007). Observing and recording copulation events of whiteflies on plants using a video camera. Èntomol. Exp. Appl..

[40] Wang T., Li L., Hong W. (2017). SNARE proteins in membrane trafficking. Traffic.

[41] Nair U., Klionsky D.J. (2011). Autophagosome biogenesis requires SNAREs. Autophagy.

[42] Itakura E., Kishi-Itakura C., Mizushima N. (2012). The Hairpin-type Tail-Anchored SNARE Syntaxin 17 Targets to Autophagosomes for Fusion with Endosomes/Lysosomes. Cell.

[43] Geng L., Qian L.-X., Shao R.-X., Liu Y.-Q., Liu S.-S., Wang X.-W. (2018). Transcriptome profiling of whitefly guts in response to Tomato yellow leaf curl virus infection. Virol. J..

[44] Li M., Zhao J., Su Y.-L. (2020). Transcriptome Analysis of Gene Expression Profiles of Tomato Yellow Leaf Curl Virus-Infected Whiteflies over Different Viral Acquisition Access Periods. Insects.

[45] Luan J.-B., Li J.-M., Varela N., Wang Y.-L., Li F.-F., Bao Y.-Y., Zhang C.-X., Liu S.-S., Wang X.-W. (2011). Global Analysis of the Transcriptional Response of Whitefly to Tomato Yellow Leaf Curl China Virus Reveals the Relationship of Coevolved Adaptations. J. Virol..

