# Supplementary material for: Bemisia tabaci Vesicle-Associated Membrane Protein 2 Interacts with Begomoviruses and Plays a Role in Virus Acquisition
Source: Cells. 2021 Jul 5;10(7):1700. doi: 10.3390/cells10071700 (PMC8306474; doi:10.3390/cells10071700)
Supplement: Supplementary file 1 [file cells-10-01700-s001.zip › Supplementary Materials.pdf]

Supplementary Materials

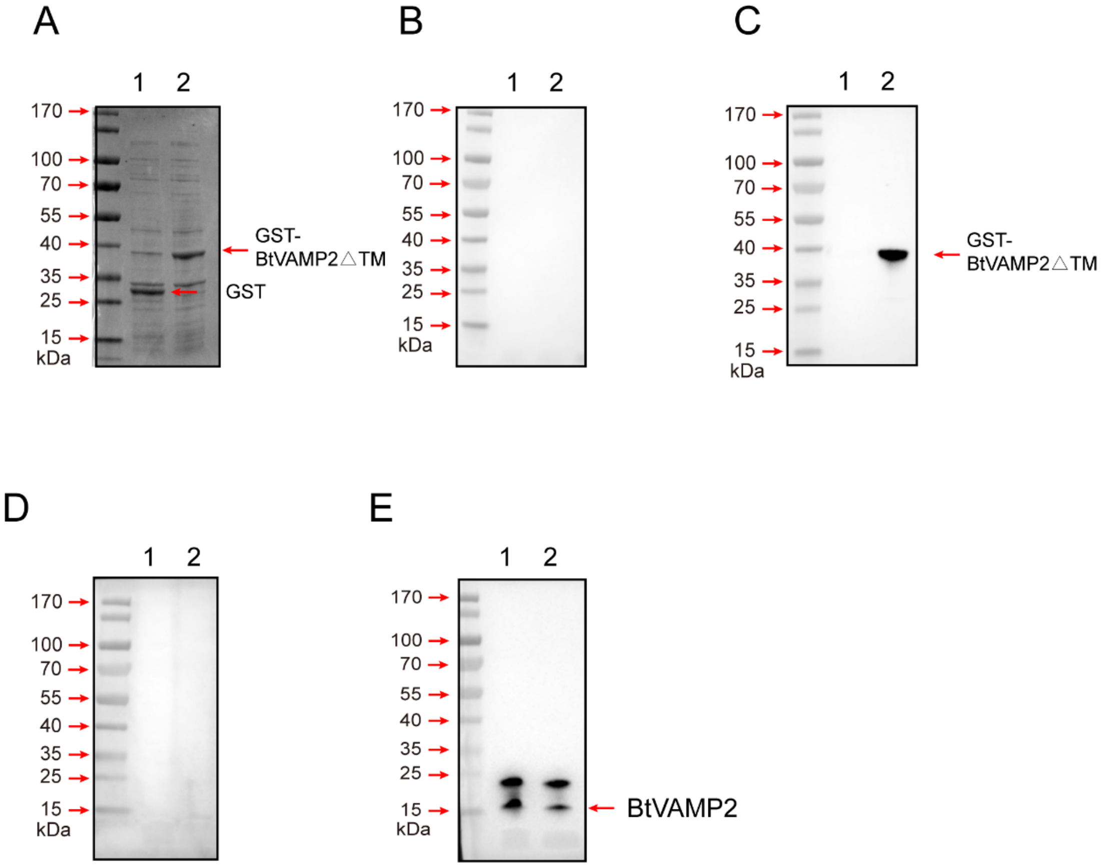

Figure S1. Analysis of anti-BtVAMP2 antibody. (A) Coomassie blue staining analysis of *Escherichia coli* (BL21) total proteins that expressed with GST protein (1) or with GST- BtVAMP2 ΔTM protein (2). The transmembrane domain of BtVAMP2 was removed for easier prokaryotic expressing. GST≈26 kDa, GST- BtVAMP2 ΔTM≈36 kDa. (B) *E. coli* (BL21) total proteins that expressed with GST protein (1) or with GST- BtVAMP2 ΔTM protein (2) were detected using rabbit pre-immune serum. (C) *E. coli* (BL21) total proteins that expressed with GST protein (1) or with GST- BtVAMP2 ΔTM protein (2) were detected using anti-BtVAMP2 rabbit pAb. (D) The proteins of MEAM1 whitefly (1) or Asia II 1 whitefly (2) were detected using rabbit pre-immune serum. (E) The proteins of MEAM1 whitefly (1) or Asia II 1 whitefly (2) were detected using anti-BtVAMP2 rabbit pAb.

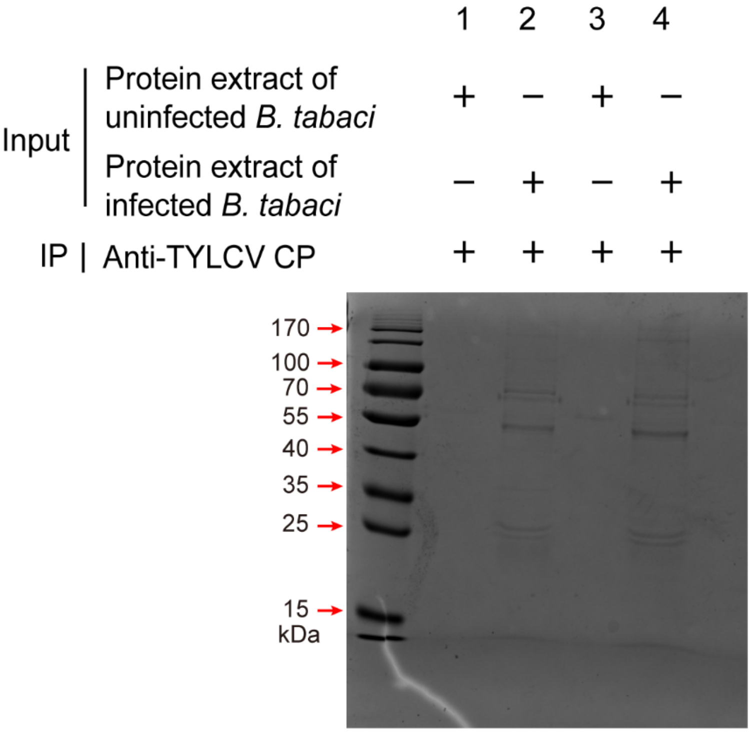

Figure S2. The candidate proteins of MEAM1 whitefly that bind to TYLCV CP by Co-IP analysis via Coomassie blue staining.

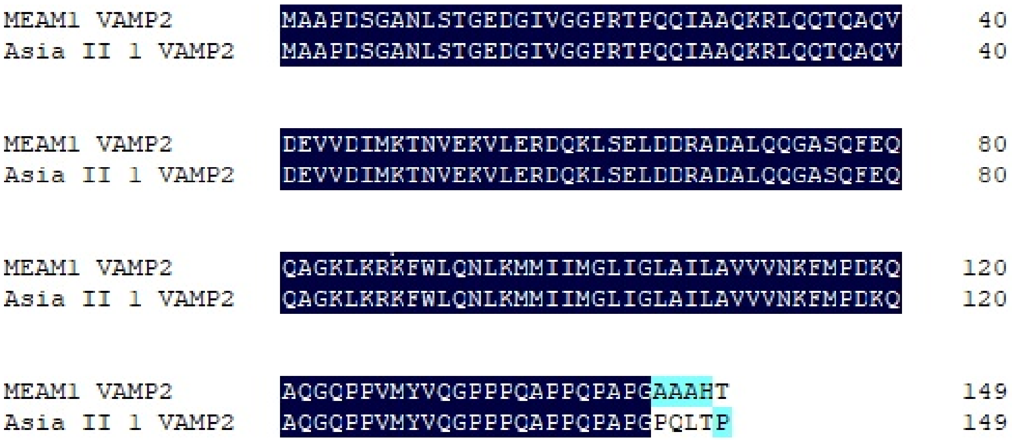

Figure S3. Comparison of BtVAMP2 amino acid sequences of MEAM1 and Asia II 1. The alignments were conducted with DNAMAN.
